# Microvascular endothelial function is blunted in postmenopausal females with frequent hot flushes

**DOI:** 10.1113/EP093733

**Published:** 2026-09-01

**Authors:** William Stokes, Chowdhury Tasnova Tahsin, Miguel F. Anselmo, Stephany Nathe, Emma J. Lee, Soliana Teshome, Marnie L. Vanden Noven, Anne Blaes, Benjamin W. Langworthy, Manda L. Keller‐Ross

**Affiliations:** ^1^ Division of Physical Therapy and Rehabilitation Science, Department of Family Medicine and Community Health, Medical School University of Minnesota Minneapolis Minnesota USA; ^2^ School of Public Health Johns Hopkins University Baltimore Maryland USA; ^3^ Department of Exercise Science Belmont University Nashville Tennessee USA; ^4^ Division of Hematology and Oncology, Medical School University of Minnesota Minneapolis Minnesota USA; ^5^ Division of Biostatistics and Health Data Science, School of Public Health University of Minnesota Minneapolis Minnesota USA

**Keywords:** cardiovascular disease, menopause, vasomotor symptoms

## Abstract

Hot flushes of menopause are associated with elevated blood pressure (BP) and diminished macrovascular endothelial function, an initiating factor in the onset of atherosclerosis. Whether microvascular endothelial function is impaired in females with hot flushes, however, remains unknown. We hypothesised that postmenopausal females with hot flushes would have reduced microvascular endothelial function and elevated BP in comparison to age‐matched females without hot flushes. Participants were healthy postmenopausal females, aged 45–70 years; participants experiencing at least three hot flushes per day were placed in the hot flush (HF) group (*n* = 16), whereas participants with no history of hot flushes were placed in the non‐HF group (*n* = 18). Heart rate (ECG) and BP (finger plethysmography) were measured during a 10 min rest period, and endothelial function was measured with pulse amplitude tonometry (EndoPAT 2000), quantified by the reactive hyperaemia index (RHI) and the natural logarithm of the RHI (LnRHI). Groups were similar in age, age at menopause and body mass index, in addition to systolic BP, diastolic BP and heart rate between groups (*P *> 0.05). The RHI was lower in the HF group (1.87 ± 0.47 a.u.) compared with the non‐HF group (2.31 ± 0.67 a.u., *P* = 0.047), and the difference between LnRHI in the HF group (0.63 ± 0.21 a.u.) and the non‐HF group was nearly significant (0.80 ± 0.27 a.u., *P* = 0.052). New findings from this study demonstrate a lower microvascular endothelial function in females with hot flushes in comparison to females without hot flushes, although BP and heart rate were not different between the groups. The reduced microvascular endothelial function demonstrated in females with hot flushes is likely to contribute to greater cardiovascular disease development.

## Abstract (highlights)

- **What is the central question of this study?**
  Do postmenopausal females who experience frequent and moderate hot flushes of menopause experience blunted endothelial function in comparison to postmenopausal females with no history of hot flushes?
- **What is the main finding and its importance?**
  Postmenopausal females who experience hot flushes of menopause and are otherwise healthy did not exhibit elevated blood pressure or heart rate but demonstrated impaired microvascular endothelial function. These findings suggest that females with hot flushes might experience impaired microvascular endothelial function and elevated cardiovascular disease risk even when presenting with a similar haemodynamic profile to those without hot flushes.

## 1 INTRODUCTION

The leading cause of death for females in the USA is cardiovascular disease (CVD) (Martin et al., 2024). Menopause, the cessation of sex hormone production, is likely to be a contributing factor because it is associated with an elevated blood pressure (BP) in older females compared with age‐matched males (Choi et al., 2017) and an increase of CVD risk factors (El Khoudary & Thurston, 2018). Indeed, several studies (Armeni et al., 2023; Franco et al., 2015; Gast et al., 2010; Jackson et al., 2016; Lee et al., 2018), but not all (James et al., 2004), demonstrate that vasomotor symptoms (VMS; hot flushes and night sweats), one of the most common menopause symptoms affecting ≤80% of peri‐ and postmenopausal females (Williams et al., 2008), demonstrate elevated BP in comparison to those without VMS (Franco et al., 2015; Gast et al., 2010). Furthermore, the frequency (Jackson et al., 2016) and severity (Zhu et al., 2020) of VMS is associated with greater CVD risk. Importantly, these relationships remain even after adjusting for traditional CVD risk factors, such as BP, waist‐to‐hip ratio or cholesterol (Gast et al., 2010; Jackson et al., 2016). Although it has been hypothesised that changes in sympathetic nerve activity might drive the relationship between VMS and CVD (Carson & Thurston, 2023), previous work in our laboratory found there was no difference in sympathetic activity in females with and without VMS (Stokes et al., 2025), although symptomology experienced in this previous study was low to moderate. Thus, alternative mechanisms might underpin the association between VMS and CVD.

One potential alternative mechanism that might drive the underlying association between VMS and CVD risk is endothelial dysfunction, characterized in postmenopausal females by decreased nitric oxide (NO)‐dependent vasodilatation (Moreau et al., 2020). This reduction in NO‐dependent vasodilatation decreases the vasodilatory buffering of the endothelium in response to vasoconstriction, thereby contributing to elevated BP (Owlya et al., 1997). Endothelial dysfunction is the initiating step in the development of atherosclerosis (Lakatta & Levy, 2003) and is associated with future CVD risk in postmenopausal females (Rossi et al., 2008). Several studies using flow‐mediated dilatation (FMD), the gold standard measure for the non‐invasive evaluation of macrovascular endothelial function (Stoner et al., 2013), have found that females with hot flushes experience a decline in endothelial function (Bechlioulis et al., 2010; Nilsson et al., 2024; Thurston et al., 2008, 2017; Witkowski et al., 2025), with some reporting declines only in those with severe hot flushes (Nilsson et al., 2024; Tuomikoski et al., 2009) and another in females ≤53 years old (Thurston et al., 2017). However, potential alterations in microvascular function, or the endothelial function of peripheral conduit vessels (Kuvin et al., 2003), have not yet been investigated in females with hot flushes.

Digital pulse amplitude tonometry (PAT), a measure of NO‐dependent vasodilatory function of the fingertips at rest and during reactive hyperaemia, quantified via the reactive hyperaemia index (RHI; Nohria et al., 2006), is associated with a number of CVD risk factors, including elevated BP (Hamburg et al., 2008, 2011; Schnabel et al., 2011). Although both endothelial PAT and FMD measure NO‐dependent pathways of endothelial function, the role of NO in these processes is distinct, playing a larger part in modulating tissue metabolism of oxygen in microvascular endothelial function and acting as a direct vasodilator in macrovascular endothelial function (Trochu et al., 2000). Furthermore, the large‐scale, longitudinal Framingham Offspring Study found that although both RHI and FMD were correlated with CVD, both measures were associated with different CVD risk factors (Hamburg et al., 2008, 2011), and there was no correlation between RHI and FMD in participants who were assessed for both outcomes (Hamburg et al., 2011). These results have led some to hypothesise that micro‐ and macrovascular endothelial function are distinct pathways to the development of atherosclerosis and that both should be evaluated when possible (Flammer et al., 2012; Hamburg et al., 2011). Furthermore, although previous studies have demonstrated the relationship between hot flushes and FMD, the relationship between microvascular endothelial function and VMS remains unknown.

Therefore, the purpose of this study was to investigate whether postmenopausal females who experience frequent and moderate hot flushes of menopause demonstrate altered microvascular endothelial function in comparison to those who have never experienced hot flushes. We hypothesised that postmenopausal females with hot flushes would demonstrate greater resting BP and lower microvascular endothelial function in comparison to postmenopausal females who have never experienced hot flushes. Furthermore, we hypothesised that among females experiencing hot flushes, the frequency and severity of hot flushes would be positively correlated with BP and negatively correlated with microvascular endothelial function.

## 2 MATERIALS AND METHODS

### 2.1 Ethical approval

This study was approved by the University of Minnesota's Institutional Review Board (IRB: STUDY00013742), registered with ClinicalTrials.gov (NCT04439370) and was conducted in accordance with the *Declaration of Helsinki*. Informed consent was obtained from all participants in writing.

### 2.2 Participants

Participants were recruited through University of Minnesota StudyFinder, social media and word of mouth. There were 469 females who were screened, and 426 of these participants were found to be ineligible. Thus, 43 participants were invited to participate in the study. During the informed consent visit, nine participants declined to continue with further study involvement. Finally, 34 postmenopausal females, between the ages of 45 and70 years old, who self‐reported experiencing either at least three hot flushes per day or never experiencing hot flushes, completed the study. A criterion of at least three hot flushes per day (Abdali et al., 2010) was chosen to ensure that our study population was symptomatic to detect potential differences in endothelial function without limiting recruitment. Participants were asked, ‘If you experience hot flashes, on average, how many hot flashes do you experience a day?’. Participants experiencing one or two hot flushes a day were excluded from the study. Postmenopausal status was determined by self‐report of ≥12 months of amenorrhoea and confirmed with estradiol levels of <30 pg/mL and follicle‐stimulating hormone (FSH) levels of ≥30 pg/mL via blood sampling (Harlow et al., 2012). Exclusion criteria for the study included: a body mass index (BMI) ≥ 35kg/m^2^; conditions known to alter autonomic function; diabetes or uncontrolled asthma; diagnosed carotid stenosis; history of CVD (excluding hypertension); nicotine/tobacco use within the past 6 months; respiratory disease; severe neurological conditions, such as stroke or traumatic brain injury; sleep apnoea; current use of menopausal hormone therapy; and use of medications that might affect heart rate, BP or autonomic function (excluding medications used to treat hypothyroidism). Use of a neurokinin‐3 antagonist to treat hot flushes was not an exclusion criterion; however, no study participant reported taking this class of medication. Participants who reported prior menopausal hormone therapy use had not received hormone therapy within the last year.

### 2.3 Experimental protocol

Participants who currently experienced at least three hot flushes per day were assigned to the HF group (*n* = 16), and participants with no history of hot flushes were assigned to the non‐HF group (*n* = 18). Visit 1 took place over Zoom, a virtual meeting platform. Participants provided informed consent, completed a medical questionnaire to ensure eligibility and completed questionnaires including: the State‐Trait Anxiety Inventory (STAI‐State and Trait) to determine anxiety levels (Spielberger et al., 1999); the Patient Health Questionnaire–9 (PHQ‐9) to evaluate symptoms of depression (Kroenke et al., 2001); and the Pittsburgh Sleep Quality Index (PSQI) and Insomnia Severity Index (ISI) to assess sleep quality (Buysse et al., 1989; Morin et al., 2011). Finally, participants completed the Menopause‐Specific Quality of Life Questionnaire (MENQOL), a survey in which participants rank on a scale of 1–8 how bothered they are by menopausal symptoms, with 1 representing the absence of the symptom, 2 representing ‘not bothered’ by a symptom and 8 indicating ‘extremely bothered’ by a symptom (Hilditch et al., 1996). Each item assesses how bothersome a symptom is in one of four domains (VMS, psychosocial, physical or sexual), and means for each subscale are computed by dividing the sum of the participant's answers by the total number of items relevant to that domain.

Visit 2 occurred at the University of Minnesota after an overnight fast and a 12 h abstention from caffeine, exercise and alcohol. Participants repeated the STAI‐State and MENQOL surveys on the morning of the study prior to the experimental procedures. Height and weight were measured to calculate BMI.

Participants were then instrumented for heart rate (HR) and BP. Heart rate was recorded via a three‐lead ECG (ADInstruments, Colorado Springs, CO, USA), and BP was recorded using a non‐invasive blood pressure (NIBP) wrist unit (ADInstruments) via finger plethysmography. A manual sphygmomanometer was used to take two baseline BP measurements to calibrate the NIBP before the 10 min rest period. If the consecutive systolic blood pressure (SBP) or diastolic blood pressure (DBP) measurements were not within 5mmHg of each other, a third BP measurement was obtained. Resting ECG and NIBP waveforms were recorded with LabChart 8 software (ADInstruments). R‐Wave peaks in the ECG were used to quantify HR, and NIBP minima and maxima were used to calculate SBP and DBP, which were subsequently used to calculate mean arterial pressure (MAP).

Participants then completed a 10 min quiet supine rest while BP and HR were recorded continuously. Following this rest period, endothelial PAT was assessed via finger blood flow from the EndoPAT‐2000 device (Itamar Medical, Israel) during a 6 min baseline, a 5 min occlusion and a 5 min postocclusion period. During the occlusion period, an SC10D Rapid Version Cuff (Hokanson, Bellevue, WA, USA) on the participant's upper left forearm was inflated via an E20 rapid cuff inflator (Hokanson) to ≥60 mmHg above their SBP. The RHI and the natural logarithm of the reactive hyperaemia index (LnRHI) were calculated automatically from the endothelial PAT recordings using EndoPat‐2000 software v.6.2 (Itamar Medical). Blood flow waveforms were inspected manually to ensure that the baseline, occlusion and postocclusion periods were identified correctly by the software and that no artefacts occurred during the areas highlighted for analysis.

Following these assessments, a blood sample was obtained to measure FSH, estrone, estradiol, total oestrogens, progesterone, testosterone, 25‐hydroxyvitamin D_3_ and haemoglobin A1c. Bloods were processed by the M Health Fairview Hospital Acute Laboratory. Liquid chromatography was used to measure testosterone and oestrogens (Zhou et al., 2017), and chemiluminescent enzyme immunoassay was used to measure FSH and progesterone (Toishi et al., 2018). Bloodwork was available for 32 participants: one participant opted out of the blood sampling procedure, and there was an error in processing the complete bloodwork of an additional participant. Of those 32 participants, progesterone levels were undetectable in 11 (*n* = 21), haemoglobin A1c levels were not processed in 2 owing to technical errors (*n* = 30), and estradiol and testosterone were undetectable in 2 different individuals (*n* = 31).

### 2.4 Statistical analysis

SPSS Statistics v.28.0.1 (IBM, Chicago, IL, USA) was used to perform all statistical analysis. To ensure the normality of data, a Shapiro–Wilk test was used. An independent *t*‐test was used to compare questionnaire results, hormone levels and resting haemodynamic and endothelial measurements between groups. Hedge's *g* was used to calculate the effect size of significant differences in haemodynamic or endothelial measurements. To determine whether there was a relationship between either the frequency of hot flushes or the severity of VMS and haemodynamic or endothelial variables, a bivariate Pearson product coefficient was calculated for both the self‐reported frequency of daily hot flushes and MENQOL‐VMS visit 2 score and all haemodynamic and endothelial function variables. Only participants experiencing VMS symptoms were included in this analysis. A sensitivity power analysis indicated that, with 16 participants in the treatment group and 18 control subjects at α = 0.05 (two‐tailed) and 80% power, the study was able to detect a between‐group difference in RHI of ∼0.58 a.u. (*g* ≥ 0.99). Results are expressed as the mean ± SD. A significance level of *P *< 0.05 was used for all analysis.

## 3 RESULTS

**FIGURE 1:**
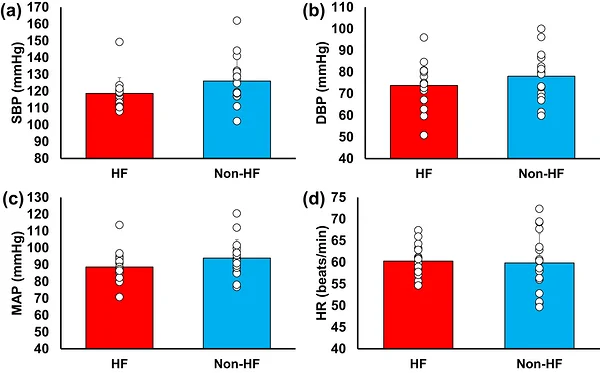
Resting haemodynamic variables in females with hot flushes (HF) and without HFs (non‐HF). (a) Systolic blood pressure (SBP) was not different between females with HFs (119 ± 9 mmHg; mean± SD) and without HFs (non‐HF; 126 ± 14 mmHg). (b) There was also no difference in diastolic blood pressure (DBP) between females with HFs (74 ± 11 mmHg) and females without HFs (78 ± 11 mmHg). (c) Mean arterial pressure (MAP) was analogous between females with HFs (89 ± 7 mmHg) and females without HFs (93 ± 11 mmHg). (d) Heart rate (HR) was similar between females with HFs (60 ± 4 beats/min) and the non‐HF group (60 ± 7 beats/min). Independent samples *t*‐tests were conducted to compare data between the HF and non‐HF groups.

**FIGURE 2:**
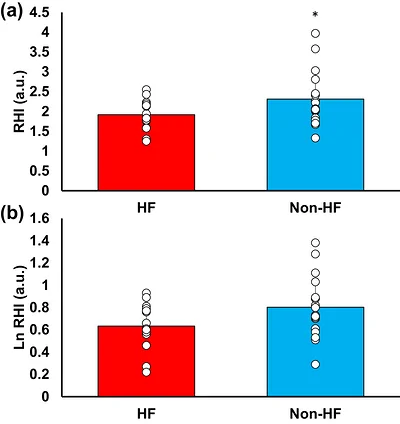
Reactive hyperaemia index (RHI) in females with hot flushes (HF) and without HFs (non‐HF). (a) The RHI was blunted in females with HFs (mean ± SD: 1.87±0.47 a.u.) compared with those without HFs (2.31 ± 0.67 a.u.). (b) There was a trend towards a blunted natural logarithm of the RHI (LnRHI) in females with HFs (0.63 ± 0.21 a.u.) compared with females without HFs (0.80 ± 0.27 a.u.). Independent samples *t*‐tests were conducted to compare data between the HF and non‐HF groups. *Significant difference between groups.

Data on participant demographics, hormone levels and questionnaire results can be found in Table 1. Data from questionnaires completed on the morning of visit 2 (STAI‐State and MENQOL) are reported owing to their temporal proximity to study visit 2 (Table 1). There were no group differences in age (*P *> 0.05) or BMI (*P *> 0.05). There were no group differences in FSH (*P *> 0.05), estrone (*P *> 0.05), estradiol (*P* = 0.60), total oestrogens (*P *> 0.05), progesterone (*P *> 0.05), testosterone (*P *> 0.05), haemoglobin A1c (*P *> 0.05) or 25‐hydroxyvitamin D_3_ (*P *> 0.05). Furthermore, there were no differences in STAI‐state (*P *> 0.05), STAI‐trait (*P *> 0.05), PHQ‐9 (*P *> 0.05), PSQI (*P *> 0.05) or ISI (*P *> 0.05) questionnaire scores between females with hot flushes and females without hot flushes. The MENQOL‐VMS score was significantly higher in females with hot flushes in comparison to females without hot flushes (*P *< 0.01), whereas there were no differences between MENQOL‐psychological (*P *> 0.05), MENQOL‐physical or MENQOL‐sexual (*P *> 0.05) subdomains. There were no group differences in resting SBP (*P* > 0.05, *g* = 0.57; Figure 1a), DBP (*P *> 0.05, *g* = 0.36; Figure 1b), MAP (*P *> 0.05; *g* = 0.42, Figure 1c) or HR (*P *> 0.05; *g* = 0.00, Figure 1d). There was a blunted RHI in the HF group compared with the non‐HF group (*P* = 0.047, *g* = 0.73; Figure 2a) and a blunted LnRHI in the HF group compared with the non‐HF group that neared statistical significance (*P* = 0.052, *g* = 0.68; Figure 2b). Finally, we did not observe a relationship between either hot flush frequency or MENQOL‐VMS domain score or in any haemodynamic or endothelial function variables (*P *> 0.05).

**TABLE 1:** Participant characteristics.

Characteristic	HF	Non‐HF	*P*‐value
Age, years	57 ± 6	60 ± 5	0.11
Age at menopause, years	47 ± 10	47 ± 11	0.98
BMI, kg/m^2^	26 ± 4	25 ± 4	0.49
STAI‐State, a.u.	30 ± 8	30 ± 8	0.85
STAI‐Trait, a.u.	30 ± 7	28 ± 8	0.38
PHQ‐9, a.u.	2 ± 3	2 ± 2	0.81
Hot flush frequency, per day	5 ± 2	n/a	n/a
MENQOL – Hot Flush score, a.u.	4 ± 1	1 ± 0	<0.01
MENQOL – Psychosocial score, a.u.	2 ± 1	2 ± 1	0.41
MENQOL – Physical score, a.u.	2 ± 1	2 ± 1	0.11
MENQOL – Sexual score, a.u.	2 ± 1	2 ± 1	0.56
PSQI, a.u.	7 ± 3	6 ± 3	0.13
ISI, a.u.	7 ± 4	6 ± 5	0.37
FSH, IU/L	91 ± 31	89 ± 23	0.85
Estrone, pg/mL	20 ± 7	27 ± 23	0.29
Estradiol, pg/mL	7 ± 6	6 ± 6	0.60
Total oestrogen, pg/mL	23 ± 10	33 ± 29	0.24
Progesterone, ng/mL	0.2 ± 0.2	0.3 ± 0.2	0.28
Testosterone, ng/dL	18 ± 5	20 ± 10	0.32
25(OH)D_3_, nmol/L	51 ± 25	39 ± 8	0.08
HbA1c, %	5.5 ± 0.3	5.6 ± 0.3	0.45

Abbreviations: 25(OH)D_3_, 25‐hydroxyvitamin D_3_; BMI, body mass index; FSH, follicle‐stimulating hormone; HbA1c, haemoglobin A1c; HF, hot flush; ISI, insomnia severity index; MENQOL, menopause quality of life questionnaire; n/a, not applicable; PHQ, patient health questionnaire; PSQI, Pittsburgh sleep quality index; STAI, state‐trait anxiety index.

Sample sizes: Age at menopause: HF, *n* = 15, non‐HF, *n* = 14; PSQI: HF, *n* = 15; non‐HF, *n* = 17; FSH, estrone, 25_OH_D3: HF, *n* = 16; non‐HF, *n* = 16; estradiol, total oestrogen, testosterone: HF, *n* = 16; non‐HF, *n* = 15; progesterone: HF, *n* = 10; non‐HF, *n* = 11; HbA1c: HF, *n* = 15; non‐HF, *n* = 15.

## 4 DISCUSSION

VMS of menopause are strongly associated with CVD, but the mechanisms driving this relationship are unclear. New findings from this study suggest that microvascular function is reduced in postmenopausal females with frequent and moderate hot flushes. Contrary to our hypothesis, however, females in the HF group did not demonstrate elevated BP compared with those in the non‐HF group. Furthermore, there were no relationships observed between the frequency or severity of hot flushes and haemodynamic variables or endothelial function. These findings suggest that microvascular endothelial function is altered in postmenopausal females with frequent and moderate hot flushes, despite sharing a similar haemodynamic profile with females who have never experienced hot flushes.

### 4.1 VMS and haemodynamic profile

Multiple studies have found elevated BP in females with VMS in comparison to those without VMS after controlling for traditional CVD risk factors (Armeni et al., 2023; Franco et al., 2015). Additionally, data from the Study of Women's Health Across the Nation (SWAN), a longitudinal study, reported that among menopause‐related factors, such as age at menopause and estradiol levels, VMS were predictive of the rise in BP throughout the menopausal transition (Samargandy et al., 2022). In contrast to this observation, several studies have demonstrated that VMS do not influence BP (Hautamäki et al., 2011; James et al., 2004; Tuomikoski et al., 2009), including our own work (Stokes et al., 2025). These studies were similar to the present study with regard to sample size and stringent exclusion criteria.

One explanation for the lack of observed differences in BP between the groups in this study is that the decline in endothelial function in females with hot flushes has not led to structural changes in the vasculature that would consequently influence BP. Consistent with our findings, the SWAN study observed macrovascular endothelial dysfunction in females with hot flushes without differences in BP or carotid intima–media thickness (Bechlioulis et al., 2010). It is feasible to suggest that the females in the present study demonstrate a decline in endothelial function prior to the onset of calcified plaques. Over time, this vascular remodelling might lead to atherosclerosis and elevated BP (Glagov et al., 1987). The timing of these changes in relationship to ageing and menopause, however, is still relatively unclear.

### 4.2 VMS and vascular function

VMS of menopause are associated with an adverse cardiometabolic profile, such as elevated BP, increased levels of low‐density lipoprotein (LDL) cholesterol and greater BMI, in comparison to individuals who do not experience VMS (Gast et al., 2010). When adjusting for these CVD risk factors, however, females with frequent and persistent VMS of menopause still have a higher incidence of CVD than females with less frequent or no VMS (Thurston et al., 2021). This finding suggests that non‐traditional CVD risk factors, such as endothelial dysfunction, might be a major driver of this elevated risk. Previous work has shown that macrovascular endothelial function declines through each stage of the menopausal transition (Moreau et al., 2012) and is further reduced in females with hot flushes (Bechlioulis et al., 2010; Nilsson et al., 2024; Thurston et al., 2008, 2017; Tuomikoski et al., 2009; Witkowski et al., 2025). Some studies demonstrate that endothelial function is impaired only in females with severe hot flushes (Bechlioulis et al., 2010; Nilsson et al., 2024), whereas others show an attenuation only in younger females (age 40–53 years) with early‐onset hot flushes (Thurston et al., 2017). Thus, there is some disagreement among studies regarding which cohort among females with hot flushes experiences the greatest decline in endothelial function in comparison to similarly aged counterparts without hot flushes. Moreover, studies thus far have used macrovascular rather than microvascular endothelial measures (Bechlioulis et al., 2010; Nilsson et al., 2024; Thurston et al., 2008, 2017; Tuomikoski et al., 2009; Witkowski et al., 2025), and the two measures might represent distinct pathways for the development of CVD (Alexander et al., 2020). The present study used endothelial PAT and demonstrated, for the first time, that females with frequent and moderate hot flushes experience impaired microvascular endothelial function in comparison to females who have never experienced hot flushes.

Although micro‐ and macrovascular endothelial function are governed by different physiological mechanisms (Climie et al., 2019), and their respective measures of RHI and FMD are either weakly correlated (Schnabel et al., 2011) or not correlated (Hamburg et al., 2011), both are reliant on NO‐dependent mechanisms (Axtell et al., 2010; Green et al., 2014). One of the primary determinants of endothelial function is the production of NO, a molecule which is synthesized from the amino acid l‐arginine by the enzyme endothelial nitric oxide synthase and released by the endothelium in response to shear stress (Förstermann & Münzel, 2006). Although the average RHI and LnRHI of both the HF and non‐HF groups is considered normal based on clinical criteria (Bonetti et al., 2004), the lower RHI and/or LnRHI demonstrated in this study suggests that postmenopausal females with hot flushes demonstrate attenuated microvascular endothelial function compared with females having no history of hot flushes.

The reduced endothelial function observed in the present study is consistent with others using FMD to measure macrovascular endothelial dysfunction in females with hot flushes (Bechlioulis et al., 2010; Nilsson et al., 2024; Thurston et al., 2008, 2017; Witkowski et al., 2025). Still, our cohort investigated microvascular endothelial function only in postmenopausal females, thus it remains unknown whether these results can be extended to perimenopausal participants. Although the mechanisms of endothelial dysfunction in females with hot flushes are not understood, there are several possible contributory factors influencing NO availability. For example, estradiol facilitates increased NO production through endothelial nitric oxide synthase and estradiol receptor‐mediated mechanisms (Nevzati et al., 2015), in addition to the downregulation of endothelin‐1 production (Tan et al., 2003), meaning that the loss of estradiol during the menopausal transition is not only the initiating factor for VMS onset, but also contributes to endothelial dysfunction. Indeed, estrogen therapy in postmenopausal females improves NO‐mediated endothelial function to premenopausal levels (Majmudar et al., 2000). Given the above studies, changes in estradiol availability might be a crucial driver in the loss of endothelial function experienced by postmenopausal females.

Although estradiol plays an important role in modulating endothelial function, we did not observe differences in estradiol, nor did we expect differences in estradiol between these two groups (Aksel et al., 1976; Freedman & Woodward, 1996). Estradiol levels were also not associated with endothelial function, suggesting that other mechanisms beyond the loss of estradiol are likely to be driving the observed differences in endothelial function in females with VMS. Elevated LDL cholesterol has been observed in females with VMS (Gast et al., 2010), which can contribute to vascular inflammation and, subsequently, endothelial dysfunction (Gliozzi et al., 2019). Previous research has found, however, that blunted macrovascular endothelial function is observed in females with hot flushes even when controlling for levels of LDL cholesterol (Thurston et al., 2008, 2017). Given these prior findings, this potential mechanism might be insufficient to explain our findings, although without measuring cholesterol, we are unable to state this definitively.

High sympathetic nerve activity, which can disrupt adipose cells and increase levels of LDL cholesterol (Geerling et al., 2014), has been shown to have an inverse relationship with RHI (Sverrisdóttir et al., 2010) and thus might influence endothelial function. In addition, there is indirect evidence of increased sympathetic activity during hot flushes via changes in adrenaline and noradrenaline levels (Cignarelli et al., 1989; Kronenberg et al., 1984), leading some to hypothesise that hot flushes might be sympathoexcitatory, which, in turn, drives the increased risk of CVD in females with VMS (Miller et al., 2018; Nilsson et al., 2024). Previous work from our laboratory has demonstrated that sympathetic activity, quantified with muscle sympathetic nerve activity, the gold‐standard measure of sympathetic function, was similar in females with and without VMS at rest and in response to a stressor (Stokes et al., 2025), although our target group demonstrated low to moderate VMS symptoms. Although no resting differences in sympathetic activity between females with and without VMS were observed, BP often declines during hot flushes (Low et al., 2008, 2011), which might cause a baroreflex‐mediated increase in sympathetic activity that, over time, could cause endothelial dysfunction and increased risk of CVD (Nardone et al., 2020). Indeed, even acute changes in sympathetic activity have been found to alter artery shear rate patterns (Padilla et al., 2010), which can influence endothelial function. Further research should investigate whether sympathetic activity increases in response to the observed reduction in BP that might take place during a hot flush (Low et al., 2008, 2011) to determine whether greater sympathetic activation during hot flushes occurs chronically and whether this greater sympathoexcitation exacerbates endothelial dysfunction.

Kisspeptin/neurokinin B/dynorphin neurons are strongly implicated in the generation of vasomotor menopausal symptoms, contributing to the thermoregulatory and cutaneous vascular responses that characterize the hot flush (Jayasena et al., 2015; Padilla et al., 2010). These hypothalamic pathways are not considered direct regulators of the systemic cardiovascular system, and the pivotal phase 3 trials of neurokinin‐3 receptor antagonists, fezolinetant (SKYLIGHT 1, 2 and 4) (Johnson et al., 2023; Lederman et al., 2023; Neal‐Perry et al., 2023) and the dual neurokinin‐1/3 receptor antagonist elinzanetant (OASIS 1 and 2) (Pinkerton et al., 2024) did not report effects of these agents on resting heart rate or BP. Nonetheless, indirect cardiovascular and sympathetic effects mediated by these pathways remain plausible and warrant dedicated investigation.

Although previous epidemiological work has found a relationship between the frequency and severity of VMS and both reduced FMD (Nilsson et al., 2024; Tuomikoski et al., 2009) and greater CVD risk (Jackson et al., 2016; Zhu et al., 2020), the mechanisms driving these associations remain unclear. One possible mechanism might be elevated plasma levels of cortisol and interleukin‐6 or other pro‐inflammatory cytokines, which are elevated in females with moderate to severe VMS (Gibson et al., 2016; Reed et al., 2016) and linked to the development of atherosclerosis (Su et al., 2021). We did not observe a relationship between either the level of hot flush bother or the frequency of hot flushes and RHI. We did not measure cortisol or pro‐inflammatory cytokines; thus, we are unable to determine whether cortisol or immune factors could explain findings in this study. Additionally, given that our study cohort was postmenopausal and has potentially been symptomatic for years, it might be that age‐related decreases in hot flush frequency and severity contributed to the null relationship between RHI and frequency of hot flushes.

### 4.3 Strengths and limitations

This study has strengths and limitations that should be considered while interpreting the data. One of the major strengths of this study is the use of a well‐validated measure of microvascular function, endothelial PAT (Bonetti et al., 2004), to observe, for the first time, that microvascular function is altered in otherwise healthy females with hot flushes. Using a stringent set of inclusion/exclusion criteria ensured that other CVD risk factors, such as age or BMI, did not confound data collected in this study population. In addition, including females with a frequency of three or more hot flushes a day was consistent with larger‐scale epidemiology studies demonstrating a relationship between hot flushes and macrovascular endothelial function (Nilsson et al., 2024; Thurston et al., 2008).

Limitations of the study should be considered when interpreting the data. First, this study is limited by a lack of information regarding the age of hot flush onset, because hot flush timing might influence functional (Thurston et al., 2017) and structural (Thurston et al., 2016) changes of macrovascular endothelial function in females with hot flushes. Second, although this study did not assess physical activity levels, previous research has found that physical activity might play a role in mediating the relationship between VMS and macrovascular function (Witkowski et al., 2025). Additionally, endothelial PAT is an indirect physiological measure derived from changes in digit pulse wave amplitude during reactive hyperaemia. Although the hyperaemia response is mediated, in part, by NO‐dependent microvascular function, this vasodilatation is a complex process, and NO‐independent mechanisms modulating digit blood flow, such as the release of adenosine (Costa et al., 1999), could be driving the results of this study (Nohria et al., 2006). Our sample was modest (HF, *n* = 16, non‐HF, *n* = 18), and a sensitivity analysis indicated that the study was powered to detect only large effects (*g* ≥ 0.99 at 80% power), while the observed difference was medium to large (*g* = 0.73). Thus, the study was underpowered relative to the observed effect. However, the primary comparison was statistically significant (*P* = 0.047). Our study also used self‐report measures of hot flushes, whereas some (Thurston et al., 2017; Witkowski et al., 2025), but not all (Bechlioulis et al., 2010; Nilsson et al., 2024; Thurston et al., 2008) studies investigating this relationship used 24 h hot flush monitoring. Future studies investigating hot flushes and endothelial function should use both self‐report measures of hot flushes and objective ambulatory recordings.

## 5 CONCLUSION

In conclusion, postmenopausal females with current moderate and frequent hot flushes demonstrated blunted microvascular endothelial function compared with females having no history of hot flushes. These findings provide insight that microvascular function, in addition to macrovascular function (Bechlioulis et al., 2010; Nilsson et al., 2024; Thurston et al., 2008, 2017), is impaired in females with hot flushes and might be driving the greater development of CVD in females who experience frequent hot flushes. Future research should investigate how endothelial dysfunction develops across the menopausal transition and to what extent it is responsible for the increased risk of CVD among females with VMS.

## AUTHOR CONTRIBUTIONS

William Stokes and Manda L. Keller‐Ross conceived and designed the research. William Stokes, Chowdhury Tasnova Tahsin, Miguel F. Anselmo, Stephany Nathe, Emma J. Lee, Soliana Teshome and Manda L. Keller‐Ross performed the experiments. William Stokes analysed the data and consulted with Benjamin Langworthy on statistical analysis. William Stokes and Manda L. Keller‐Ross interpreted results of the experiment. William Stokes prepared the figures and drafted the manuscript. All authors edited the manuscript and William Stokes and Manda L Keller‐Ross revised the manuscript. All authors approved the final version of manuscript and agree to be accountable for all aspects of the work in ensuring that questions related to the accuracy or integrity of any part of the work are appropriately investigated and resolved. All persons designated as authors qualify for authorship, and all those who qualify for authorship are listed.

## CONFLICT OF INTEREST

No conflicts of interest, financial or otherwise, are declared by the authors. Abstracts using some of these data were presented at the American Autonomic Society International Symposium in 2023 and the Menopause Society Annual Meeting in 2024.

## GENERATIVE AI STATEMENT

No AI tool has been used in the making of this manuscript.

## Data Availability

The data supporting the findings of this study will be made available by the corresponding author upon reasonable request.
